# Segmentation of distal airways using structural analysis

**DOI:** 10.1371/journal.pone.0226006

**Published:** 2019-12-19

**Authors:** Debora Gil, Carles Sanchez, Agnes Borras, Marta Diez-Ferrer, Antoni Rosell

**Affiliations:** 1 Comp. Vision Center and Comp. Science Dept, UAB, Barcelona, Spain; 2 Pneumology Unit, Hosp. Univ. Bellvitge, IDIBELL, CIBERES, Barcelona, Spain; 3 Hosp. Univ. Germans Trias i Pujol, Badalona, Spain; University at Buffalo, UNITED STATES

## Abstract

Segmentation of airways in Computed Tomography (CT) scans is a must for accurate support of diagnosis and intervention of many pulmonary disorders. In particular, lung cancer diagnosis would benefit from segmentations reaching most distal airways. We present a method that combines descriptors of bronchi local appearance and graph global structural analysis to fine-tune thresholds on the descriptors adapted for each bronchial level. We have compared our method to the top performers of the EXACT09 challenge and to a commercial software for biopsy planning evaluated in an own-collected data-base of high resolution CT scans acquired under different breathing conditions. Results on EXACT09 data show that our method provides a high leakage reduction with minimum loss in airway detection. Results on our data-base show the reliability across varying breathing conditions and a competitive performance for biopsy planning compared to a commercial solution.

## Introduction

Bronchoscopy examinations are the diagnostic cornerstone for lung cancer since they allow biopsy of nodules with minimum risk for the patient. A main limitation of flexible bronchoscopy is the difficulty to determine the best pathway to peripherial lesions. Physician’s accuracy at defining proper 3D routes is only around 40% for lesions located near airways at generation 4 at most, with errors beginning as early as generation 2 [1, 2]. Despite recent advances, new endoscopy techniques only increase diagnostic yield to 70% and still radiate the patient. Diagnostic yield could be improved reducing radiation and costs if imaging technologies could better guide the bronchoscopist to the target lesion.

Virtual bronchoscopic navigation (VBN) systems [3] are used to reconstruct computed tomography (CT) data into three-dimensional representations of the tracheobronchial tree. VBN systems allow for coupling virtual and real-time bronchoscopy, which is useful for guiding ultrathin bronchoscopes and other devices in diagnostic interventions [4]. Due to limited extraction of the airways from the CT data, the potential of VBN is often limited in the most peripheral regions of the lungs [5]. In a recent communication [6], segmentations not reaching the peripheral pulmonary lesions were observed in 44% of cases and in such cases, only 35% of them were diagnosed. This diagnostic rate is comparable to that achieved without a navigation software and, thus, a VBN system does not represent any advantage unless segmentations reach the most distal airways.

The segmentation of most distal bronchi is challenging because, even using high resolution (0.6 × 0.6 × 0.5 mm) CT scans, their caliber in the scanned volumes just covers a few voxels and their wall is reduced to 1-2 faint intensity voxels. This requires running segmentation algorithms using extreme values for their parameters (usually thresholds) that increase the presence of artifacts, such as leakage, in segmented airways. Leakage causes segmentations to extend outside the airway and leak into the lung parenchyma [7]. To avoid leakage and other artifacts while optimizing thresholds, several strategies have been proposed.

For methods based on region growing [8–12] an option is to iteratively increase the intensity threshold used to separate air from tissue while controlling the number of voxels added between consecutive segmentations. In [7] trachea, right and left bronchi are segmented independently using three different thresholds optimized to be efficient across different CT scan acquisition parameters. Another option [13–15] is to use graph structural analysis to reconstruct the bronchial tree from a set of branches obtained after thresholding of a map of bronchi local appearance. A graph is used to represent the connectivity of candidate branches and best connections are selected from a global graph-partitioning algorithm based on a cost and benefit scoring of connections [13]. Finally, a very recent work [16] uses convolutional neural networks to remove leakage from a given segmentation using local appearance. Segmentations are partitioned into segments that are classified as leakage or bronchi using a convolutional network. This classification allows the combination of segmentations computed using different thresholds.

### Contribution

All methods reviewed above base segmentations on functions (given, for instance, by convolution with filters or the probability of a classifier) that have high values at voxels belonging to airways. It follows that segmentations are defined as voxels achieving values above a given threshold. Such threshold can be set either heuristically or learned from a training set using a classifier. In any case, threshold values are global values equal for all voxels and scans and set according to local appearance, regardless of the global geometric structure of the resulting segmentation.

In this paper we present a novel method able to set patient-specific thresholds locally adapted for each airway level according to, both, bronchi local appearance and segmentation global structure. Under the ground that airways anatomy follows a tree structure, we encode segmentations using a directed graph and compute a measure of how much the graph deviates from a tree. This measure is used to locally adapt thresholds and prune segmentations artifacts.

To show the potential of our graph structural analysis, we also present a strategy for the segmentation of most distal airways. Our strategy is based on a thresholding of a map of airways local appearance computed by convolution with an own-designed tubular filter in a multiresolution scheme to account for differences in airways caliber. We call this method PICASSO: PerIpheral bronChiAl Segmentation with Structural Optimization. We present results on the EXACT09 database [17] and on an own series of high resolution CT scans acquired at Hospital de Bellvitge under different breathing conditions [18].

## Materials and methods

Our method is based on energy maps (describing airways appearance) binarized using a threshold adapted for each case. Thresholds are computed to ensure that segmentations have a shape with optimal match to airways anatomical structure and minimum leakage. The matching criterion uses a measure of anatomical consistency based on the complexity of a graph representation of the segmented airways. Zero complexity is associated to segmentations without leakage, while leakage volume increases along with complexity positive values. In case the graph is computed for the whole segmentation, we obtain a global threshold able to provide a leakage free initial segmentation. In case the graph is computed for a subvolume only containing a distal branch, the threshold based on graph complexity is adapted according to the branch local structure. Further, if thresholding is restricted to each branch surroundings, any initial segmentation can be independently refined for each distal branch. Finally, the same structural analysis based on graph complexity provides an algorithm for the suppression of leakage which might be applied as a post-processing filtering step in case that thresholds are computed for a positive complexity.

In the next Sections we explain our measure of anatomical consistency (Section Structural Analysis for Anatomically Consistent Segmentations) and the main steps of the proposed segmentation strategy (Section Strategy for Segmentation of Distal Airways).

### Structural analysis for anatomically consistent segmentations

For any map, *E* = *E*(*i*, *j*, *k*), such that airway voxels have higher values than background voxels, a segmentation of airways, *Seg*_*Th*_, can be obtained by thresholding as:

$$\begin{array}{c} Seg_{Th}\left(i,j,k\right)=\{\begin{array}{ccc} 1 & \;\text{if}\; & E(i,j,k)>Th\\ 0 & \;\text{otherwise} \end{array} \end{array}$$ (1)

In our case the map *E* is obtained (see Section Airways Local Appearance Maps) from the convolution with a bank of filters describing bronchi local appearance in CT-scans. Thus, from now one, these maps will be called appearance maps.

We set an optimal *Th* according to the following measure of the consistency of *Seg*_*Th*_ geometry with airways anatomy. Airways are tubular structures with their geometry determined by the centerline given by bronchi lumen center. These centerlines have a tree structure given by bronchi branching levels. In order to quantify segmentations anatomical consistency we analyze the geometry of their skeleton. To do so, the segmentation skeleton is encoded in a graph that represents its branching geometry by nodes and edges. The nodes of the graph correspond to skeleton branching points and its edges represent branch connectivity.

The trachea entry point allows directing the graph using Depth First Search (DFS). In the absence of artifacts, the directed graph should be a (binary) tree with levels corresponding to bronchial levels and leafs corresponding to the most distal points achieved by the segmentation. In practice, segmentations might include structures and artifacts not belonging to bronchial anatomy which alter the graph tree structure.

A connected graph is a tree if and only if it is cycle-free. A directed graph is cycle-free if and only if for all leafs, there is only one path to the root. This last condition allows to measure how much a directed graph deviates from a tree, and localize what distal branches have more artifacts in their segmentation. Let $\#Pth_{Leaf_{i}}$ be the number of paths from the i-th Leaf node, *Leaf*_*i*_ to the graph root and *N*_*Leaf*_ the number of leafs, then our measure of complexity is defined as:

$$\begin{array}{c} Complexity=1-\frac{N_{Leaf}}{\sum_{i=1}^{N_{Leaf}}\#Pth_{Leaf_{i}}}\in \left[0,1\right] \end{array}$$ (2)

A tree has *Complexity* = 0, while *Complexity* approaches to 1 as the number of cycles increases.

The complexity (2) provides scan-sensitive thresholds ensuring segmentations conforming with bronchial anatomy and either free of artifacts (*Complexity* = 0) or with a controlled amount of them. Let *MxComplexity* be the maximum deviation from a tree allowed for a segmentation, *Seg*_*Th*_, and let us define a function of *Seg*_*Th*_ complexity depending on *Th* as:

$$\begin{array}{ccc} & & F_{Complex}=F_{Complex}\left(Th\right)≔\\ & & =Complexity(Seg_{Th})-MxComplexity \end{array}$$ (3)

then the threshold that achieves the maximum complexity *MxComplexity* is a zero of *F*_*Complex*_. We note that, assuming that the map *E* is the likelihood that a voxel belongs to an airway and *Seg*_*Th*_ given by (1), the zeros of *F*_*Complex*_ are also given by:

$$\begin{array}{c} ThOpt≔min_{Th}\left(F_{Complex}\left(Th\right)<=0\right) \end{array}$$ (4)

The computation of the optimal threshold requires finding a zero of the complexity function *F*_*Complex*_ defined in (3). Since it is continuous and bounded in the range [1 − *MxComplexity*, −*MxComplexity*], the equation can be solved using any iterative numerical method for the resolution of non-linear equations. In particular, we could use the bisection method, since Bolzano’s Theorem [19] ensures its convergence to one solution provided that the function is continuous and achieves opposite signs in each bound of the search interval. Bolzano’s method can be implemented using the iterative algorithm described in Algorithm 1. Given that convergence to a solution of *f*(*x*) = 0 is guaranteed, the algorithm can be stopped when a given accuracy for the solution, tolerance *tol* in Algorithm 1, is achieved. In our case, *f* = *F*_*Complex*_, *xa*, *xb* are defined by a minimum and maximum threshold values (see Section Experimental Design for a rule to set them) of the map *E* and *xc* = *ThOpt*.

**Algorithm 1** Pseudo Code of Bolzano’s bisection method for the resolution of non-linear equations

*xc* = (*xa* + *xb*)/2

*yc* = *f*(*xc*)

**while** *abs*(*yc*) > *tol* **do**

 **if** *ya* * *yc* < 0 **then**

 | *xb* = *xc*

 **end**

 **else**

 | *xa* = *xc*

 **end**

 *xc* = (*xa* + *xb*)/2

 *yc* = *f*(*xc*)

**end**

The solution to *F*_*Complex*_ = 0 set a global threshold equal for all voxels in case (2) is computed from the graph associated to the complete segmentation *Seg*_*Th*_. In case the graph is computed for a subvolume only containing a distal branch, its complexity exclusively depends on the local structure of the segmented branch. Therefore the solutions to (3) provide thresholds locally adapted to such bronchial branch (see Section Distal Refinement of Initial Segmentation for further details).

In case *MxComplexity* is positive, some distal subtrees might contain artifacts in their segmentation. Such artifacts are detected as cycles in the graph associated to the segmentation and are pruned as follows. For each cycle, their nodes are sorted according to increasing values of *E* to be iteratively removed until the complexity of the graph is zero. The collection of all nodes removed together with their edges defines a subset of the segmentation 3D skeleton. The inverse skeletonization of this subset provides a volume that contains the artifacts attached to the segmented distal subtree. The complementary of the volume of artifacts in *Seg*_*ThOpt*_ is our final segmentation with artifact reduction.

### Strategy for segmentation of distal airways

**Fig 1:**
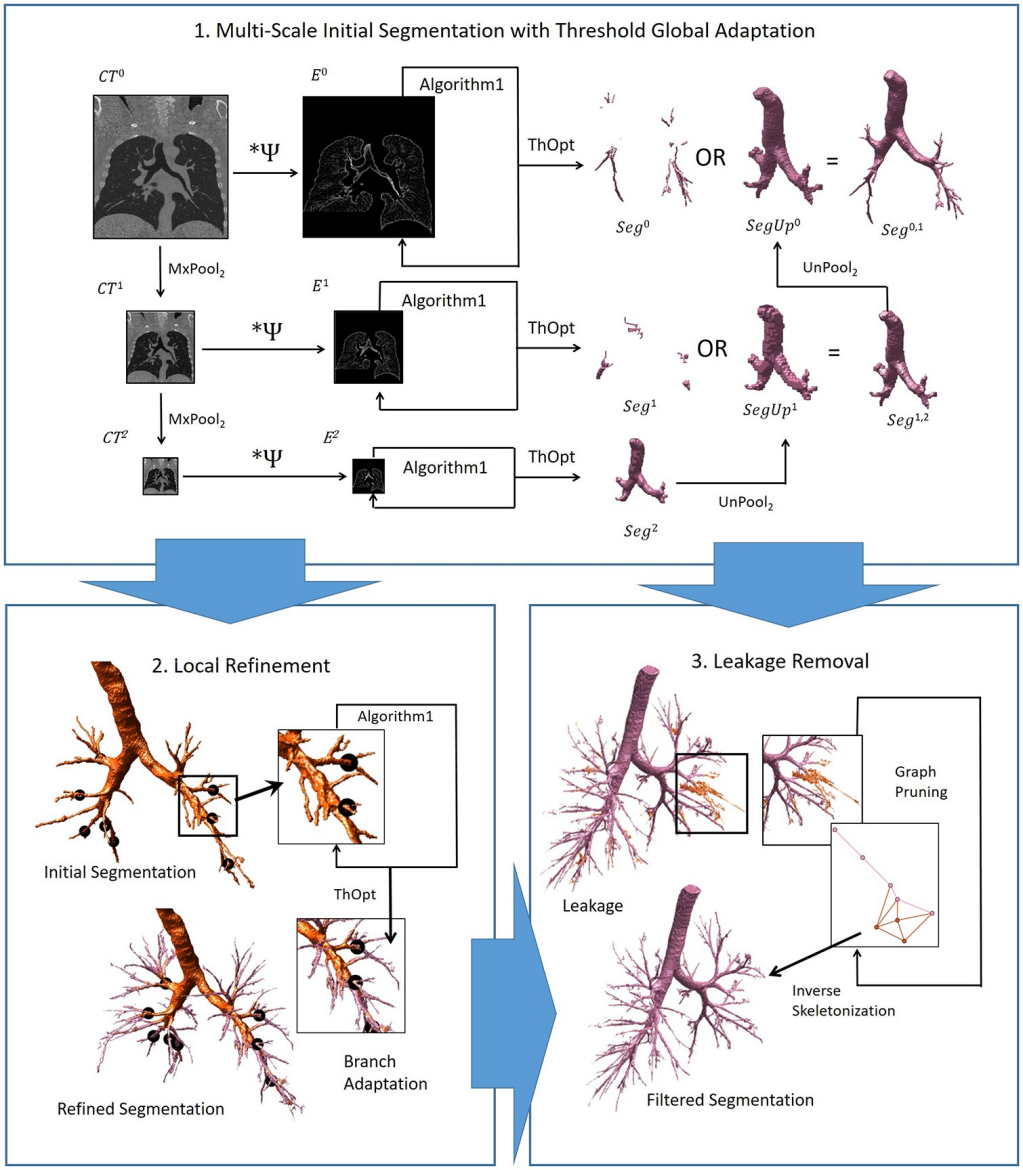
PICASSO main steps. 1. Initial segmentation using a multi-scale approach with global adaptation of the threshold. The input data is the original CT scan (left image) and the output are airway segmentations (most right meshes shown in purple) at different scales, one for each down-scaled CT volume. 2. Distal refinement of initial segmentation (upper left orange mesh) with a threshold locally adapted for each distal branch (marked with dark circles). The output refined distal branches are shown in purple in the lower left mesh. 3. Leakage removal by pruning the cycles of the graph representing the segmentation skeleton. The input segmentation with leakage is shown in orange (both in the upper left mesh and graph), while the output filtered leakage-free segmentation is shown in purple.

Our strategy PICASSO is based on a thresholding of bronchi local appearance maps. Appearance maps are computed by convolution with a bank of own-designed tubular filters, while threshold values are adapted using the graph structural analysis of Section Structural Analysis for Anatomically Consistent Segmentations. Fig 1 shows a flowchart with the main steps of our segmentation method PICASSO.

First, we compute an initial segmentation of main bronchi using a multi-scale approach with global adaptation of the threshold for each scale. Our tubular filtering is applied at each down scaled volume, labelled *CT*^*l*^, obtained by repeated pooling of the original gray-scale CT volume. The size of the kernel used in tubular filtering is the same for all scales and it is set to the scale of distal bronchi at full resolution. By performing a pooling, followed by a filtering at a fix small scale, we can detect airways of different levels and sizes without increasing the kernel size [20]. For each pooled volume at scale *l*, its appearance map, namely *E*^*l*^, is binarized with the threshold that solves (3) for *F*_*Complex*_ computed using *E*^*l*^. For each such a map at scale *l*, the optimal threshold is computed by applying Algorithm 1 to *F*_*Complex*_ computed from *E*^*l*^.

Second, we refine the initial segmentation at full resolution with local adaptation of the threshold according to the consistency of each distal branch. To do so, we compute a graph for subvolumes that only contain each distal branch, so that the threshold based on graph complexity computed by applying Algorithm 1 is exclusively obtained according to the branch local structure. The subvolumes are computed using a binary mask of the surroundings of each distal branch and the thresholding is also restricted to such surroundings. This way the initial segmentation can be independently refined for each distal branch.

Finally, leakage removal could be applied at any of the former steps in case we allow for a drop in consistency in the computation of the thresholds. This is controlled by setting a positive value for the tolerance parameter *MxComplexity* in Eq (3).

#### Pre-Processing

CT scans are first pre-processed to in-paint [21] pulmonary vessels and body tissue outside lungs in order to suppress responses at such interfaces prone to introduce artifacts. In-painting requires the segmentation of the volume regions that have to be in-painted.

Body tissue is defined as the complementary of lungs in CT scans. To segment lungs and pulmonary vessels we apply a threshold to CT scans Hounsfield units. Before thresholding, scans are convolved with a bank of 3D anisotropic Gaussian kernels in order to homogenize Hounsfield values. Let *σ* = (*σ*_*x*_, *σ*_*y*_, *σ*_*z*_) be the scale of the filter and Θ = (*θ*, *ϕ*) its orientation given by the unitary vector *η* = (*cos*(*ϕ*)*cos*(*θ*), *cos*(*ϕ*)*sin*(*θ*), *sin*(*ϕ*)), then an oriented anisotropic 3D Gaussian kernel, $g_{\sigma }^{\Theta }$, is given by:

$$\begin{array}{c} g_{\sigma }^{\Theta }=g_{\sigma }^{\Theta }\left(x,y,z\right)=\frac{1}{{\left(2\pi \right)}^{3/2}\sigma_{x}\sigma_{y}\sigma_{z}}e^{-(\frac{{\tilde{x}}^{2}}{2\sigma_{x}^{2}}+\frac{{\tilde{y}}^{2}}{2\sigma_{y}^{2}}+\frac{{\tilde{z}}^{2}}{2\sigma_{z}^{2}})} \end{array}$$ (5)

for $\left(\tilde{x},\tilde{y},\tilde{z}\right)$ the change of coordinates given by the rotations of angles *θ* and *ϕ* that transform the z-axis into the unitary vector *η*. The convolution of the CT scan with the Gaussian kernel enhances areas of homogeneous intensity, like air in lungs, trachea and main bronchi (negative responses) and blood in pulmonary vessels (positive responses). Lungs and vessel are segmented applying Otsu thresholding to the negative response to an isotropic Gaussian kernel for the lungs and the positive response to a bank of anisotropic Gaussian kernels for pulmonary vessels.

The in-painting inside the segmented structures is based on a nearest neighbor interpolation of CT intensity values. The in-painted volumes, denoted by *CT* = *CT*(*i*, *j*, *k*), are the input for the computation of bronchi local appearance maps.

#### Airways local appearance maps

The filter describing bronchi local appearance is given by a blob detector customized to detect tubular structures. The blob detector is given by a Laplacian operator, $\Delta_{\sigma }^{\Theta }$ computed using the 2nd derivatives of the Gaussian kernel (5) as:

$$\begin{array}{ccc} \Delta_{\sigma }^{\Theta } & & =\Delta_{\sigma }^{\Theta }\left(x,y,z\right)=\partial_{xx}g_{\sigma }^{\Theta }+\partial_{yy}g_{\sigma }^{\Theta }+\partial_{zz}g_{\sigma }^{\Theta }=\\ & & =\left({\tilde{x}}^{2}/\sigma_{x}^{4}+{\tilde{y}}^{2}/\sigma_{y}^{4}+{\tilde{z}}^{2}/\sigma_{z}^{4}\right)g_{\sigma }^{\Theta }-\left(1/\sigma_{x}^{2}+1/\sigma_{y}^{2}+1/\sigma_{z}^{2}\right)g_{\sigma }^{\Theta } \end{array}$$ (6)

with the scales set to *σ*_*z*_ > > *σ*_*y*_ = *σ*_*x*_ in order to detect tubular-like anisotropic structures. We crop the filter along its long axis (*z*-axis) to obtain a filter consistent with a tubular shape. The cropping planes are set at z-levels including the most negative values of (6), so that our tubular kernel, denoted by $\psi =\psi_{\sigma }^{\Theta }$, is given by:

$$\begin{array}{c} \psi =\psi_{\sigma }^{\Theta }=\{\begin{array}{cc} \Delta_{\sigma }^{\Theta } & \left|z\right|\leq \tilde{z}\\ 0 & \;\text{otherwise} \end{array} \end{array}$$ (7)

for $\tilde{z}$ the value such that the left tail of a 1D gaussian distribution given by $g_{\sigma }^{\Theta }\left(0,0,\tilde{z}\right)$ is under 0.01. The kernel *ψ* is normalized to have unitary *L*^2^ norm and centered to have zero integral. The convolution of the CT scan with *ψ* enhances airways tubular structures (positive responses) and airways walls (negative responses).

The maximum positive response to a bank of oriented anisotropic tubular filters defines the local appearance maps of, both, main and distal airways provided that filter scales are adapted to the size of main and distal airways.

#### Initial segmentation of main bronchi

Main bronchi up to 4-6 level have very different calibers. Instead of convolving CT volumes with filters of varying scales, we adopt a multi-scale approach and repeatedly down-sample the original volume. For each down-sampling scale, denoted by *l*, we convolve the scaled volume with a bank of filters of fixed size. For each such convolution, the appearance map, noted *E*^*l*^, is binarized using a global threshold, denoted by *ThOpt*^*l*^, adapted to the scale using Algorithm 1 for *F*_*Complex*_ computed from *E*^*l*^ binarizations.

The volume *CT* is subsequently down-sampled by a factor of 2 using a max pooling operator [22]. Max-pooling was preferred to smoothing operators (like average or median) for the sake of the preservation of the highest contrast voxels and under the assumption that partial volume attenuation of bronchial borders is over noise. A max pooling of window size *SzePool* = *H* × *W* × *D* and stride (*S*_*h*_, *S*_*w*_, *S*_*d*_) = (*H*, *W*, *D*) is given by:

$$\begin{array}{ccc} & & \;MxPool_{SzePool}\left(V\right)\left(i,j,k\right)=\max_{i^{\prime },j^{\prime },k^{\prime }}V\left(i+\left(i^{\prime }-1\right)S_{h},j+\left(j^{\prime }-1\right)S_{w},k+\left(k^{\prime }-1\right)S_{d}\right)\\ & & \;1\leq i^{\prime }\leq H,1\leq j^{\prime }\leq W,1\leq k^{\prime }\leq D \end{array}$$

for *V* = *V*(*i*, *j*, *k*) denoting any 3D volume. In our case, since *H* = *W* = *D* = 2, we will note *MxPool*_*SzePool*_ by *MxPool*_2_.

Using the above notation, the down-scale volumes, denoted by *CT*^*l*^, are subsequently computed from the original scans, *CT* = *CT*^0^, as:

$$\begin{array}{c} CT^{l}=MxPool_{2}\left(CT^{l-1}\right) \end{array}$$ (8)

for *l* = 1, …, *L*, being *L* the maximum number of poolings.

For each level of down-sampling, *CT*^*l*^ is convolved with a bank of oriented filters $\psi_{\sigma }^{\Theta }$ with the scale *σ* fixed for all levels. The maximum response to such filter bank defines the appearance map *E*^*l*^ that characterizes bronchi at the level of detail given by the down-sampling. These maps *E*^*l*^ are computed as:

$$\begin{array}{ccc} & & \;E^{l}=\underset{\Theta }{\text{max}}\left(CT^{l}*\psi_{\sigma }^{\Theta }\right) \end{array}$$ (9)

For each scale, *l*, we applied Algorithm 1 to *F*_*Complex*_ computed from *E*^*l*^ in order to obtain optimal thresholds, noted by *ThOpt*^*l*^, globally adapted to each scale. Let *Seg*^*l*^ denote the segmentation at the scale *l* obtained by thresholding *E*^*l*^ with *ThOpt*^*l*^:

$$\begin{array}{c} Seg^{l}=Seg_{ThOpt}^{l}\left(i,j,k\right)≔\{\begin{array}{ccc} 1 & \;\text{if}\; & E^{l}\left(i,j,k\right)>ThOpt^{l}\\ 0 & \;\text{otherwise} \end{array} \end{array}$$

then, the multiresolution segmentation scheme we propose is given by:

$$\begin{array}{ccc} & & \;Seg^{l-1,l}≔\max\left(Seg^{l-1},SegUp^{l}\right))\;l=1,\ldots ,L\\ & & \;Seg^{L,L+1}≔Seg^{L} \end{array}$$

with *SegI* ≔ *Seg*^0,1^ and *SegUp*^*l*^ denoting the un-pooling of *Seg*^*l*^ given by:

$$\begin{array}{c} \begin{array}{c} \;UnPool_{SzePool}\left(V\right)\left(i^{\prime },j^{\prime },k^{\prime }\right)=MxPool\left(V\right)\left(i,j,k\right)\\ S_{h}\left(i-1\right)\leq i^{\prime }\leq S_{h}\left(i-1\right)+H\\ S_{w}\left(j-1\right)\leq j^{\prime }\leq S_{w}\left(j-1\right)+W\\ S_{d}\left(k-1\right)\leq k^{\prime }\leq S_{d}\left(k-1\right)+D \end{array} \end{array}$$ (10)

In our case, *H* = *W* = *D* = 2, so that *UnPool*_*SzePool*_ = *UnPool*_2_. We observe that the max/un-pooling operation on segmented volumes given by (10) can be understood as an OR operation between two increasing pooling levels. If the coarser level *l* is above the threshold, all the neighboring voxels of the finer level *l* − 1 are forced to belong to the segmentation. Otherwise, only those above threshold in the finer scale *l* − 1 (if any) will be included.

#### Distal refinement of initial segmentation

In order to refine the initial segmentation using thresholds locally adapted to each distal branch, tubular filtering and its binarization required to optimize (4) are restricted to a Region of Interest (ROI) containing each distal branch of the initial segmentation, *SegI*. Such ROIs are defined as binary masks computed from *SegUp*^1^ and *SegI* as follows.

Let *SegI*^*c*^ denote the complementary mask of *SegUp*^1^ in *SegI* given by *SegI*^*c*^ ≔ *SegI* \ *SegUp*^1^. The connected components of this mask are a collection of *SegI* most distal branches. For each such component, denoted by *SegIB*_*cc*_, consider its complementary in *SegI*, $SegIB_{cc}^{c}≔SegI\backslash SegIB_{cc}$. Then, the ROI mask, namely *ROI*_*cc*_, containing exclusively the distal component *SegIB*_*cc*_ is computed from *SegIB*_*cc*_ and $SegIB_{cc}^{c}$ distance maps as:

$$\begin{array}{ccc} & & ROI_{cc}≔\{\begin{array}{ccc} 1 & \;\text{if}\; & d\left(SegIB_{cc}\right)\leq d\left({\left(SegIB_{cc}\right)}^{c}\right)\\ 0 & \;\text{otherwise} \end{array} \end{array}$$

for *d*(⋅) denoting the 3D distance map to a volume mask.

The intersection between *E*^0^ and *ROI*_*cc*_ defines an appearance map, *E*_*cc*_ ≔ *E*^0^ ⋂ *ROI*_*cc*_ which cancels outside *ROI*_*cc*_. This way the graph encoding *E*_*cc*_ binarization only represents the local anatomy of the segmented distal branch and, thus, the optimization of its complexity provides a threshold adapted to such branch.

Finally, we recall that in case that we set *MxComplexity* > 0 to optimize either global or local thresholds, we apply the leakage removal strategy as a post-processing filtering step.

### Experimental design

We carried out 2 experiments to validate our method. In Experiment1 we run our method on the EXACT09 database for comparison to existing segmentation methods. In Experiment2 we use our own CT data acquired under different breathing conditions to compare to a commercial software (LungPoint^®^, Broncus, USA) for VBN [4].

Computations were performed using a serial code implemented in MATLAB (MathWorks^®^, Natick, MA, USA) run on a Fujitsu using a Intel(R) Xeon(R) CPU E5-1620 v3 @ 3.50GHz with a Titan X Pascal to accelerate the computation of convolutions.

In order to convert segmentations to a graph, the skeleton of the segmented CT volume was obtained using [23] which is an automatic algorithm for computing subvoxel precise skeletons of volumetric data based on subvoxel precise distance fields. The skeletonization algorithm uses as input a subvoxel precise distance field and employs a number of fast marching method propagations to extract the skeleton at subvoxel precision. The skeleton is converted into a network (undirected) graph describing the segmented bronchi anatomy by nodes and edges using [24]. The input of the method [24] is a 3D binary image containing a one-dimensional voxel skeleton, generated e.g. using [23], as well as, the output is the adjacency matrix of the graph, and the nodes and links of the network as MATLAB structure.

### Parameter settings

Table 1 lists PICASSO’s parameters with a brief description, a criterion for their selection and the values used in these experiments. PICASSO has to set parameters for the optimization of segmentation’s complexity (*MxComplexity*, and Bolzano’s stop tolerance, *tol*, and search interval limits, [*x*_*a*_, *x*_*b*_] = [*Th*_*Mn*_, *Th*_*Mx*_]), the computation of appearance maps (filter bank parameters, *σ*, Θ) and the multi-scale approach maximum pooling levels, *L*.

**Table 1:** PICASSO parameters.

Parameter	Description	Selection	Values
*MxComplexity*	deviation from airways anatomy	high values increase detection and computational cost (leakage removal)	PICASSO_*B*_,0 PICASSO_*L*_,0 PICASSO,0.15
*tol*	Bolzano’s stopping criterion	*ThOpt* accuracy	50 units of appearance maps
[*Th*_*Mn*_, *Th*_*Mx*_]	Bolzano’s search interval	opposite sign of *F*_*Complex*_	[750, 975]
*σ*	tubular filter scale	distal bronchi size and scan resolution	(1,1,4)
Θ	tubular filter orientations	filters included in *σ* kernel support	Θ^*n*,*m*^ = (*nπ*/6, *mπ*/6)
*L*	multiresolution pooling levels	reduce main bronchi to distal size	3

The parameter *MxComplexity* measures the deviation of the segmentations from airways anatomy and, thus, high values increase detection of distal airways at the cost of a higher computational cost required to remove leakage. In our first experiment, we used two different complexity values and threshold selection approaches in order to illustrate its impact in segmentations. Regarding Bolzano’s stopping parameter in Algorithm 1, it is related to the accuracy of the optimal threshold and we heuristically fixed it to achieve a compromise between segmentation improvement and algorithm efficiency. Finally, the minimum, *Th*_*Mn*_, and maximum, *Th*_*Mx*_, thresholds defining Algorithm 1 search interval, should satisfy that *F*_*Complex*_(*Th*_*Mn*_) > 0 and *F*_*Complex*_(*Th*_*Mx*_) < 0. We learned this values from EXACT09 training set as the minimum (*Th*_*Mx*_) and maximum (*Th*_*Mn*_) values such that *F*_*Complex*_ > 0 and *F*_*Complex*_ < 0, for all cases in the training set.

The scale of appearance maps was the same for all resolutions and was set to detect most distal bronchi to a size including bronchi and parenchyma. Orientations were discretized so that the rotated kernels are included in the discrete support of an unrotated kernel. This size is 33 × 33 × 33 voxels and is given by the volume containing 99% of the unrotated tubular filter.

Finally, the number of multiresolution levels, *L*, was set to 3 to ensure that main bronchi have a similar size than distal ones and, thus, can be detected with tubular filters of scale (1, 1, 4).

### Data sets

#### Experiment1 (EXACT09 challenge)

Our method was applied to the 20 testing CT scans of the MICCAI Challenge EXACT09 [17] (http://image.diku.dk/exact/) acquired with different acquisition conditions including variable slice thickness (0.5-1.0 mm), in-plane voxel sizes (0.55-0.78 mm) and radiation dose (120/140 kVp, 10.0-411.5 mAs).

For evaluation we considered the reference set and metrics presented in [17] and offered by the EXACT09 site. For the reference segmentations, experienced observers manually evaluated the results offered by the 15 algorithms compared in the original competition to construct a common reference standard. Concerning quantitative metrics we considered false positive rate (artifacts), leakage volume, branches detected (count, percentage) and length (absolute and in percentage) of the detected bronchial tree.

#### Experiment2 (CPAP study)

This data set was acquired at Hospital de Bellvitge in a clinical study [18] to compare the quality of airway segmentations from CT acquisitions performed both in end-inspiration and end-expiration with different continuous positive airway pressure (CPAP) protocols (available at http://iam.cvc.uab.es/portfolio/cpap-study-database/). Scans were obtained from patients undergoing study of pulmonary lesions and referred for a diagnostic thoracic CT scan. For each patient 4 CT acquisitions of the entire lung were obtained including inspiration, expiration, inspiration with CPAP and expiration with CPAP. Scans were acquired with a 320-detector row device with slice thickness and interval of 0, 5 and 0, 4 mm respectively performed with Aquilion ONE (Toshiba Medical Systems, Otawara, Japan) with a 80 x 0.5 mm collimator, tube voltage of 100 kVp. Since this data set has not any annotation nor ground truth for segmentations, we used the number of segmented branches as validation metric.

### Statistical analysis

#### Experiment1 (EXACT09 challenge)

PICASSO’s EXACT09 metrics were compared to the metrics EXACT09 top performers. PICASSO’s EXACT09 metrics were compared to the metrics EXACT09 top performers. Top performers were selected as those methods having tree length detected above 50%. The teams selected were Team2 (automated), Team4 (automated), Team5 (automated), Team7 (automated), Team13 (automated), Team14 (automated) and Team15 (interactive). For each metric, descriptive statistics as computed by EXACT09 challenge were considered.

In order to illustrate the benefits of our adaptive thresholding and leakage removal based on graphs, we applied our method using 3 different configurations. The first one is a PICASSO base line (labelled PICASSO_*B*_) computed using a common global threshold learned from EXACT training set to achieve zero complexity, *MxComplexity* = 0. This configuration sets the maximum amount of branches that can be detected without artifacts using a fixed threshold. The second configuration (labelled PICASSO_*L*_) is a PICASSO with adaptive local threshold and *MxComplexity* = 0.15 to include high number of distal branches. This configuration sets the maximum number of branches that the tubular kernel of Section Airways Local Appearance Maps is able to detect. The last configuration (labelled PICASSO) is the full methodology with adaptive local threshold and leakage removal. Like PICASSO_*L*_, complexity was set to *MxComplexity* = 0.15 to assess the capability for leakage reduction and distal branch preservation.

#### Experiment2 (CPAP study)

For the CPAP-Study data was managed and analyzed using software R version 3.2.5. A descriptive statistical analysis was carried out for number of airways, noted by *NAir*, using the number of samples, mean and standard deviation (SD). Main analysis was performed using generalized mixed models in logarithmic scale. In particular, we followed the model that was used in the CPAP study [25] with an extra variable for the segmentation method. The CPAP model was designed by the Statistical Service of Hospital Universitari de Bellvitge.

The regression model included as factors the acquisition protocol (EXP, for expiration and INS for inspiration) and the segmentation method (LungPt/PICASSO) and a random subject effect to account for intra-individual variability among patients. Inspiratory acquisitions segmented with the commercial software LungPoint were considered the reference baseline. Measured lung volume (in mm3 as estimated by the CT scan software) was used as adjusting factor to correct for variations in respiratory maneuvers [26]. The regression model was:

$$\begin{array}{c} log\left(NAir_{ij}\right)=\beta_{0}+\beta_{1}Vol_{ij}+\beta_{2}Seg_{Mth}+\beta_{3}I+Pat_{i}+\epsilon_{ij} \end{array}$$ (11)

for *Pat*_*i*_ ∼ *N*(0, *σ*_*Pat*_) denoting the random effect that models intra-patient variability, *Vol*_*ij*_ lung volume and *SegMth*, *I* two grouping factors for, respectively, the segmentation method (*Seg*_*Mth*_ = 0 for LungPt, *Seg*_*Mth*_ = 1 for PICASSO) and the acquisition protocol (*I* = 0 for expiration and *I* = 1 for inspiration).

Model assumptions were validated by means of residual analysis and influential values. We computed model coefficients, p values and 95% confidence interval (CI) for significance in main effects. The 95% CI for the difference LungPt-PICASSO was also computed. CIs were back transformed to the original scale for their interpretation. In original scale differences between segmentation methods are expressed as a ratio. A ratio of 1 indicates an expectation that the outcome of the methods is not different. A ratio greater than 1 indicates an expected improvement (a better performance) of PICASSO relative to baseline LungPt. A ratio less than 1 indicates an expected worse performance of PICASSO. A p value < 0.05 was considered statistically significant.

## Results

### Experiment1: EXACT09 challenge data set

**Fig 2:**
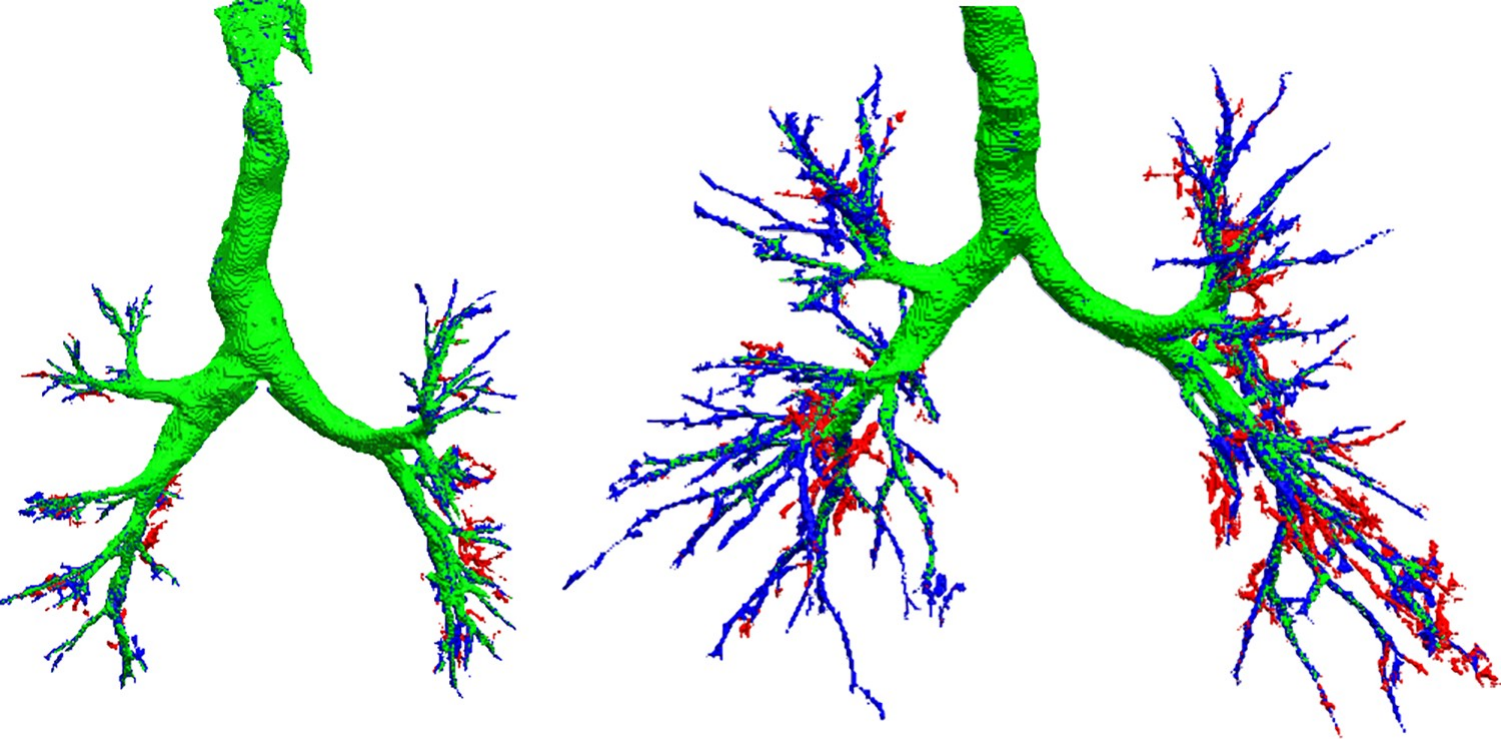
EXACT09: Performance of PICASSO in terms of leakage suppression (red voxels) and branch detection with varying complexity (complexity 0, green voxels; complexity 0.15, blue voxels). Case 24 in the left panel and case 34 in the right one.

Fig 2 shows the 3 PICASSO configurations in different colors. The baseline segmentation PICASSO_*B*_ is shown in green, PICASSO_*L*_ leakage is shown in red and PICASSO with leakage removal is shown in blue. We note that PICASSO graph structural analysis is able to remove large amounts of leakage while keeping the majority of distal branches.

**Fig 3:**
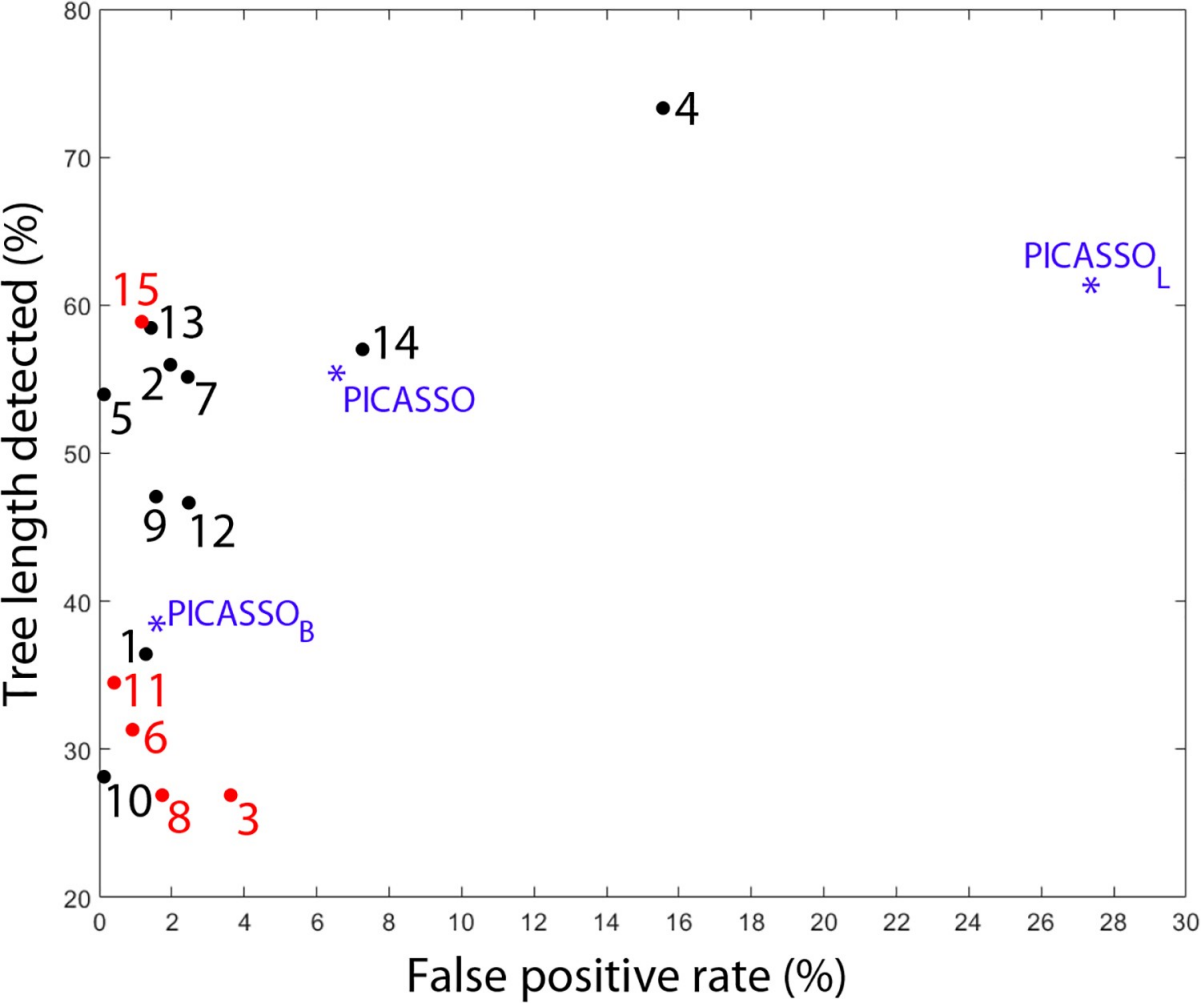
EXACT09: False positive rate vs tree length detected. The 15 bullets are results represented in [17] after the 15 algorithms originally tested, while the 3 asterisks are relevant to the 3 versions of the proposed PICASSO algorithm, which are newly inserted for a comparison.

Fig 3 shows the average tree length versus average false positive rate of all EXACT algorithms including the 3 different configurations of our method: PICASSO_*B*_, PICASSO_*L*_ and the full method with leakage removal PICASSO. Table 2 shows descriptive statistics (as reported by EXACT09) for the results obtained by the 3 PICASSO configurations and top teams mean results at the bottom rows.

**Table 2:** EXACT09: PICASSO experiments vs top teams.

	Branch count	Branch (%)	Tree length (cm)	TLD (%)	Leak count	Leak (mm^3^)	FPR (%)
PICASSO_*B*_	103.5	43.8	74.5	36.8	7.5	179.3	1.58
PICASSO_*L*_	164.9	68.0	131.8	61.4	103.7	3804.9	27.34
PICASSO	151.6	62.3	118.9	55.4	73.2	987.3	6.52
Team2	158	62.8	122.4	56	12	563	2.0
Team4	187	76.5	158.7	73.3	35	5138	15
Team5	150	59.8	118.4	54.0	2	18	0.1
Team7	147	57.9	125.2	55.2	6	577	2.4
Team13	151	63.0	122.4	58.4	5	372	1.4
Team14	161	67.2	115.4	57.0	44	1873	7.2
Team15	149	63.1	119.2	58.9	10	159	1.2

The baseline PICASSO_*B*_ has TLD = 43.8% and FPR = 1.58%. This numbers are comparable to the ones obtained by most newer methods (available at http://image.diku.dk/exact/newresults.php) evaluated after the challenge. In spite of setting *MxComplexity* = 0, PICASSO_*B*_ has FPR>0%. We think this is due to two main factors: EXACT09 metrics and using a fixed threshold learned on EXACT training set. On one hand, EXACT09 metrics base on a ground truth created from all results submitted to the original challenge. This means that if a method detects a (part of) a branch that was not detected by any of the submitted methods it will be counted as false positive [17]. These makes metrics of all methods evaluated after 2009 to be overstated in FPR and understated in TLD. On the other hand, due to variability across cases, a threshold having FPR = 0% on the training set, might include some leakage on (EXACT test set) new cases.

The configuration PICASSO_*L*_ with adaptive threshold and positive complexity, increments TLD 24.2% at the cost of a high increase (a 25.76%) in FPR. After leakage suppression (PICASSO), FPR drops 20.82% (which represents 76% of leakage removal) while 92% of branches are preserved with only 5.7% decrease in TLD. We observe that compared to EXACT top performers PICASSO is competitive in terms of airway detection (being the 3rd best in terms of % of detected branch and tree length in cm) and fair in terms of leakage presence (even good in terms of mm^3^).

### Experiment2: CPAP-Study data set

Descriptive statistics and model adjustment for the number of airways is given in Table 3. Both factors, segmentation method and inspiration, were significant (p-val < 0.001). In particular, PICASSO significantly (p-val < 0.001) increased the number of airways 1.3-fold over LungPt with a 95% CI for rate ratio equal to (1.26, 1.32). As expected the number of airways in inspiration was significantly higher than in expiration, with an average 1.7-fold increase.

**Table 3:** CPAP-Study: Model for number of airways.

Explicative variables	Descriptive			Model		
	n	mean	SD	coeff	p-val	95% CI
SegMth						
LungPoint	64	190	117	1	-	(134, 213)
PICASSO	64	239	172	0.17	<0.01	(159, 252)
Inspiration						
INS	64	305	152	1	-	(198, 327)
EXP	64	124	69	-0.65	<0.01	(103, 171)

**Fig 4:**
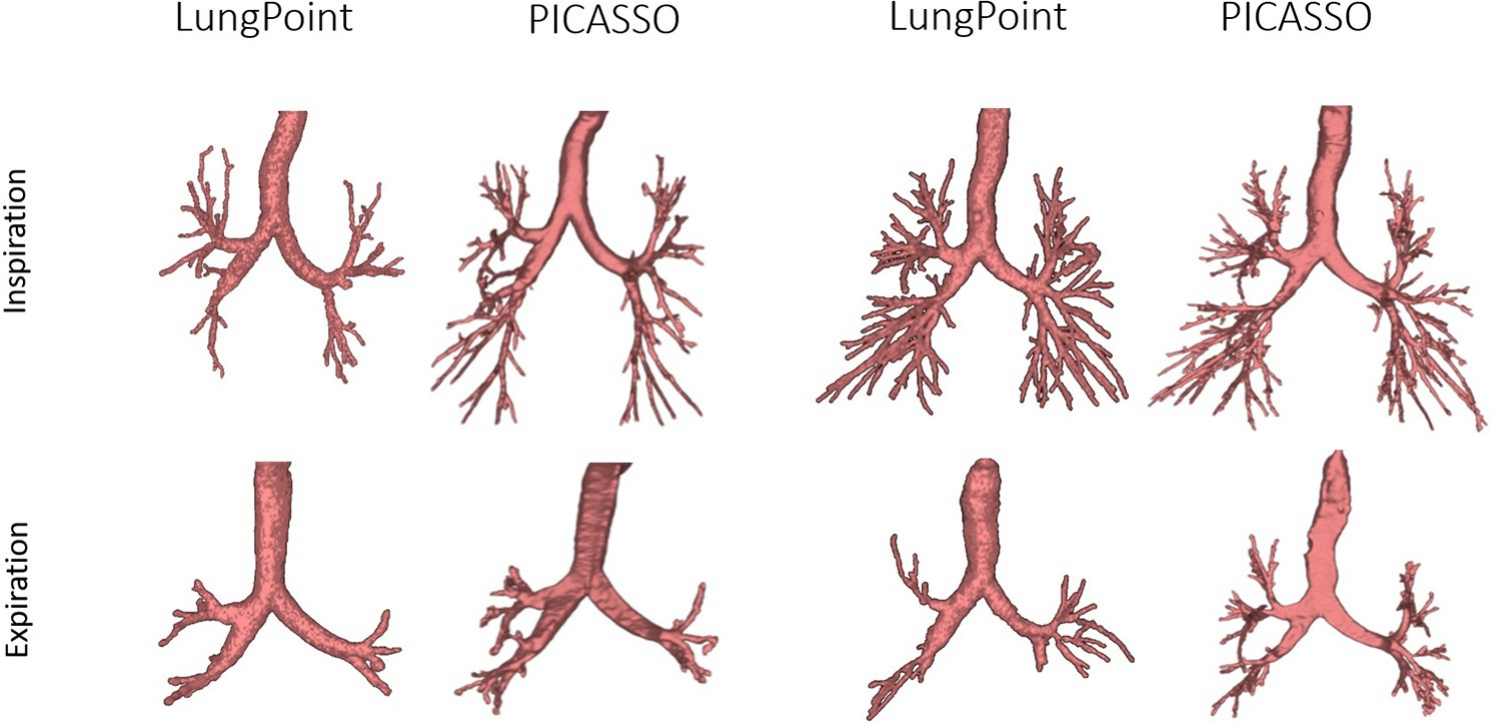
CPAP-Study:PICASSO vs LungPoint.

Fig 4 shows segmentations obtained for LungPt and PICASSO for two cases in inspiration and expiration. For both methods, the number of branches in inspiration is larger than in expiration. PICASSO segments more branches than LungPt for the inspiration of the upper case and the expiration of the lower case, whereas is comparable in the remaining cases.

## Discussion

Evaluation on EXACT09 cases shows that our method for leakage removal is able to reduce leakage 76% in average while keeping 92% of the detected branches. Comparing to top EXACT methods, PICASSO achieves a relatively high branch detection rate TDL = 62.3%, although still keeps FPR = 6.2%. Although TDL is relatively far from top performers evaluated on EXACT, like Team 4 (TDL = 76.5%; FPR = 15%) or the newer [15] (TLD = 71.6%; FPR = 9.75%) and [27] (TDL = 79.9%; FPR = 11.92%), PICASSO has a substantially lower FPR. We would like to note that metrics are bounded by the underlying method for airway detection. In this case, the multi-resolution scheme based on hand-craft filters has (PICASSO_*L*_) a top TDL = 68% with FPR = 27.34%.

Comparing to recent methods for threshold adaptation [7] evaluated on EXACT09 cases, PICASSO is superior in terms of detection but inferior in terms of leakage presence. The increase in airway detection could be attributed to different approaches for the detection of airways (multiresolution appearance maps in our case versus region growing in the case of [7]) rather than to the threshold optimization process. Also, as the same authors admit, to avoid leakage segmentation in some cases the algorithm may stop too early, avoiding possible segmentation of peripheral branches.

Comparing to newest methods for leakage removal [16] also evaluated on EXACT09 cases, our graph strategy is fairly inferior in terms of leakage removal but superior in terms of branch preservation. Experiment3 in [16] on EXACT Team14 (TDL = 59%; FPR = 7.13%) report a drop of 6.12% in FPR (20.82% for PICASSO) and a drop of 7.2% in TDL (5.7% for PICASSO). These numbers represent a 85% (76% for PICASSO) of total leakage removal with a 87.8% (91.6% for PICASSO) of detections preserved.

Concerning the computational complexity, Table 4 reports the ranges (*μ* ± *σ*) for the 2 databases (EXACT and CPAP), as well as, average scan resolution and number of cases. We report the complexity for each of the main steps of our algorithm: 1. Multi-scale initial segmentation with global threshold adaptation; 2. Distal refinement with local threshold adaptation (*PICASSO*_*L*_); and, 3. Distal refinement leakage removal (*PICASSO*). The highest computational cost is in the computation of the segmentation skeleton required for threshold optimization. In the case of the global adaptation (Step1), since the computation of the skeleton is based on fast marching, its complexity increases with scan resolution. In the case of the local refinement (Step2), given that distal branches are sequentially processed, complexity also depends on their number and, thus, it also increases along with scan resolution.

**Table 4:** Computational complexity ranges for each of the main steps of the algorithm.

Database	EXACT	CPAP
Resolution	0.69 × 0.69 × 0.95	0.5 × 0.5 × 0.5
Step 1 (min)	5.32 ± 6.03	8.16 ± 2.97
Step 2 (min)	5.26 ± 6.98	12.60 ± 3.82
Step 3 (min)	0.29 ± 0.20	0.60 ± 0.38
Cases	40	40

## Conclusion

Segmentation of most distal airways is a must for virtual bronchoscopy biopsy guiding [28]. The variability in appearance of distal regions across patients suggests using thresholds adapted for each patient and bronchial level. This paper presents an original strategy based on graph structural analysis for selecting optimal thresholds of maps codifying bronchi local appearance.

Results show that graph structural analysis can provide interesting approaches to anatomical modelling and pattern analysis without the need of exhaustive training. By incorporating anatomical structure information to segmentation methods, it is possible to achieve optimal specificity (leakage presence). Even if results are not superior in terms of sensitivity to current state-of-art supervised techniques, structural methods do not need annotated data for their design (training). Given the difficulty to produce high quality annotated clinical data, this is a main advantage for the development of clinical support systems.

The analysis of complexity in Table 4 shows that PICASSO has an average overall cost of 10.87 minutes for high resolution scans and 21.36 minutes for very high resolution scans using a MATLAB serial implementation. On one hand, such times could drop using a parallel implementation. On the hand, we observe that this complexity is not so critical for a clinical use, since airway segmentation is mainly used off-line during intervention planning.

Still, PICASSO could be improved in three aspects: leakage characterization, baseline method for airways detection and computational cost. First, the description of leakage structure as a graph cycle excludes tubular leakage that might appear parallel to airways. Although these are rare cases, they represent a portion of PICASSO 6.2% FPR and, thus, they should be also characterized to complete our measure of complexity. Second, PICASSO could improve its performance if the baseline method for airway detection was more sensitive to airways, like EXACT Team4. In this context, we think that the combination of our graph structural analysis with a baseline method based on self-learned kernels (like CNNs) could provide an optimal approach to distal branch segmentation. Although it is not a critical point for clinical use, the method complexity would significantly improve if the local refinement was parallelized to process several branches together.
